# Effect of the angiotensin-receptor-neprilysin inhibitor in heart failure patients with left ventricular ejection fraction higher than 40%: Retraction

**DOI:** 10.1097/MD.0000000000017868

**Published:** 2019-10-25

The authors have requested a retraction of the article, “Effect of the angiotensin-receptor-neprilysin inhibitor in heart failure patients with left ventricular ejection fraction higher than 40%”^[1]^ which appears in Volume 98, Issue 39 of *Medicine*. The authors have stated that there were mistakes in the methodology that make the conclusion of the article unreliable.
